# Roles and mechanisms of halophilic/salt-tolerant microorganisms in mitigating plant salt stress and promoting growth

**DOI:** 10.3389/fmicb.2026.1848153

**Published:** 2026-10-01

**Authors:** Zugen Cheng, Ping Rong

**Affiliations:** 1 Department of Cardiology, Kunming Children’s Hospital, Kunming, Kunming, China; 2 Department of Pediatrics, General Hospital of Northern Theater Command, Wenhua Road, Shenhe District, Shenyang, China

**Keywords:** halophilic microorganisms, ion homeostasis, plant growth-promoting rhizobacteria, saline agriculture, salinity stress, salt-tolerant microorganisms

## Abstract

Soil salinization is a major constraint on global agricultural productivity and food security, largely because excess salts disrupt osmotic balance, nutrient acquisition, ion homeostasis, and redox stability in plants. Halophilic and salt-tolerant microorganisms, including plant growth-promoting rhizobacteria, endophytes, and fungi, have emerged as promising biological tools for improving crop performance in saline environments. This review synthesizes current evidence on how these microorganisms mitigate salt stress and promote plant growth across diverse crop systems. The major mechanisms include modulation of phytohormone homeostasis, especially through auxin production and ACC deaminase activity; enhanced nutrient acquisition via nitrogen fixation, phosphate solubilization, and siderophore production; maintenance of ionic balance through reduced Na+ toxicity and improved K+/Na+ homeostasis; reinforcement of antioxidant defense systems; stimulation of osmolyte accumulation; and effective rhizosphere colonization through exopolysaccharide secretion and biofilm formation. Representative studies in rice, wheat, maize, tomato, soybean, cucumber, chickpea, common bean, quinoa, and banana consistently show improvements in germination, biomass, physiological resilience, and, in some cases, yield under salinity stress. However, important challenges remain, including strain specificity, inconsistent field performance, limited mechanistic resolution, ecological safety concerns, and the difficulty of translating laboratory strains into stable commercial products. Future progress will depend on multi-omics-guided mechanism dissection, precision microbiome engineering, improved formulation and delivery systems, and rigorous long-term field validation. Overall, halophilic/salt-tolerant microorganisms represent a promising strategy for saline agriculture and sustainable crop production.

## 1 Introduction

Soil salinization represents one of the most severe abiotic stresses facing global agricultural production, with its adverse effects directly impacting food security and sustainable agricultural development (Shilev, 2020). Salt stress severely inhibits plant growth, development, and yield through multiple pathways, including induction of osmotic imbalance, ion toxicity, nutrient uptake disruption, and Oxidative stress (Roy et al., 2021). Although traditional soil remediation and crop breeding strategies show some effectiveness, they are often characterized by high costs, long timeframes, or environmental risks. Therefore, developing environmentally friendly and cost-effective technologies for remediating salinized soils and enhancing crop stress resistance is crucial. Against this backdrop, utilizing microbial resources from the plant rhizosphere and endophytes—in particular, halophilic/salt-tolerant plant growth-promoting rhizobacteria (PGPR) and endophytes—has emerged as a highly promising research direction (Sagar et al., 2022; Kumar et al., 2023).

Halophilic/salt-tolerant plant growth-promoting rhizobacteria constitute a group of beneficial microorganisms that survive in high-salinity environments while colonizing the plant rhizosphere or interior (AbuQamar et al., 2024). These microorganisms intrinsically tolerate salt stress and further assist host plants in adapting to and resisting salt stress through multiple direct and indirect mechanisms, thereby promoting plant growth in saline environments (Shilev, 2020). These microorganisms are widely distributed in salt-affected lands, coastal zones, and rhizosphere microecosystems of halophytes, representing valuable biological resources endowed by nature (Mahmud et al., 2023; Jhuma et al., 2021). Recent studies demonstrate that inoculating halophilic/salt-tolerant bacteria or fungi significantly enhances seed germination rates, seedling vigor, biomass, and final yield in various crops including rice (Sagar et al., 2020; Sampangi-Ramaiah et al., 2020; Shan et al., 2023), wheat (Ayaz et al., 2022; Amini Hajiabadi et al., 2021; Thakur and Yadav, 2024), corn (Ali et al., 2022; Ali et al., 2023), tomato (Patani et al., 2023; Masmoudi et al., 2021; Yan et al., 2024), and soybean (Khan et al., 2021; Yasmin et al., 2020), while simultaneously improving plants’ physiological and biochemical states through enhanced photosynthesis, ion homeostasis regulation, osmolyte accumulation, and antioxidant defense system activation (Dey et al., 2021; Taj and Challabathula, 2021).

This mini-review reviews the core mechanisms through which halophilic/salt-tolerant microorganisms alleviate plant salt stress and promote growth, and summarizes research progress and applications across different crop systems. Building on this foundation, we explore academic controversies and research gaps in the field, and propose potential future directions to provide theoretical references for efficient and safe microbial technology applications in saline agriculture (Table 1). Several recent reviews have already catalogued the diversity, mechanisms, and molecular approaches of halotolerant rhizobacteria and their contribution to soil fertility and salinity tolerance (Sagar et al., 2022; Kumar et al., 2023; AbuQamar et al., 2024). Rather than re-cataloguing strain-crop combinations, the present mini-review is intended to complement these largely descriptive syntheses by foregrounding the unresolved controversies, the critical research gaps, and—most importantly—the persistent gap between laboratory promise and reliable field performance, thereby clarifying the conditions under which the reported benefits do and do not hold.

**Table 1:** Representative studies on the application of halophilic/salt-tolerant microorganisms for alleviating plant salt stress and promoting growth.

Crop system	Representative microorganisms or approach	Dominant mechanisms	Main outcomes under salt stress	Key references
Rice	Halotolerant rhizobacteria, endophytic fungi, and composite biofertilizers	ACC deaminase activity; N fixation; ion homeostasis; antioxidant defense; stress-responsive gene regulation	Improved seed germination, seedling vigor, nutrient status, and yield in saline soils	(Mahmud et al., 2023; Sagar et al., 2020; Sampangi-Ramaiah et al., 2020; Shan et al., 2023; Fatima et al., 2020; Khumairah et al., 2022; Saleem Asif et al., 2023)
Wheat	Salt-tolerant Bacillus/Planococcus strains, EPS-producing biofilm-forming consortia, and microbial biostimulants	Phytohormone regulation; phosphate solubilization; EPS-mediated Na + exclusion; biofilm-assisted colonization; antioxidant enhancement	Enhanced germination, biomass, grain yield, forage quality, and reduced oxidative and ionic injury	(Ayaz et al., 2022; Amini Hajiabadi et al., 2021; Thakur and Yadav, 2024; Meena et al., 2020; Muhammad Shoib Nawaz et al., 2020)
Maize	*Bacillus mycoides* PM35, halotolerant PGPR, and PGPR-based combinational treatments	Improved photosynthesis; antioxidant defense; stress-responsive gene induction; osmolyte accumulation; ROS homeostasis	Alleviated growth inhibition and improved physiological performance and yield under salinity	(Ali et al., 2022; Ali et al., 2023; Azeem et al., 2022; Liu et al., 2022; de Carvalho Neta et al., 2024)
Tomato	Bacillus spp., Bacillus spizizenii FMH45, Enterobacter 64S1, Pseudomonas 42P4, and microbial consortia	Auxin and other plant growth-promoting metabolites; antioxidant defense; osmoprotectant accumulation; ionic balance	Increased biomass, improved physiological traits, better soil fertility, and enhanced salt tolerance	(Patani et al., 2023; Masmoudi et al., 2021; Yan et al., 2024; Chintan Kapadia et al., 2021; Pérez‐Rodriguez et al., 2022; Sahu et al., 2023; Sánchez et al., 2023; Wu et al., 2024; Vaishnav et al., 2020)
Soybean	Halotolerant Pseudomonas and Bacillus, including systemic tolerance-inducing strains	Ion transport regulation; antioxidant activation; secondary metabolite modulation; osmotic adjustment	Improved growth, reduced salt-induced damage, and stronger systemic tolerance responses	(Khan et al., 2021; Yasmin et al., 2020; Yang Y. et al., 2020; Wang et al., 2023)
Cucumber	Halotolerant bacterial isolates and desert-adapted fungal endophytes/AMF-based co-inoculation	Osmotic adjustment; antioxidant enzyme induction; plant growth promotion	Improved seedling growth and better tolerance to salinity and concurrent abiotic stress	(Hosseyni et al., 2021; Fotoohiyan et al., 2024)
Chickpea	ACC deaminase-positive halophilic Bacillus isolates and biofilm-forming Bacillus spp.	Hormonal regulation; ionic and redox homeostasis; osmolyte accumulation; biofilm formation	Higher yield, lower oxidative damage, and improved adaptability in saline soil	(Nagaraju et al., 2021; Haroon et al., 2023; Basu et al., 2023)
Common bean	Exopolysaccharide-producing halotolerant Rhizobium azibense	Ion homeostasis; antioxidant defense; osmolyte accumulation; EPS-mediated protection	Improved growth and physiological adaptability under saline conditions	(Shahid et al., 2024)
Quinoa	*Burkholderia phytofirmans* PsJN and halotolerant bacteria from extreme environments	Osmotic adjustment; antioxidant enhancement; growth promotion	Improved growth and yield under salt stress	(Yañez-Yazlle et al., 2021; Yang Y. et al., 2020)
Banana	Mangrove-derived halotolerant Bacillus and Pseudomonas strains	Reduction of ROS accumulation and electrolyte leakage	Enhanced growth and physiological stability of tissue-cultured seedlings	(Abdussabur et al., 2022)

ACC, 1-aminocyclopropane-1-carboxylate; AMF, arbuscular mycorrhizal fungi; EPS, exopolysaccharides; PGPR, plant growth-promoting rhizobacteria; ROS, reactive oxygen species.

## 2 Core mechanisms of halophilic/salt-tolerant microorganisms in promoting plant growth and alleviating salt stress

Halophilic/salt-tolerant microorganisms operate in the plant root microenvironment through complex, interrelated mechanisms that enhance salt tolerance and promote plant growth at multiple levels.

Firstly, regulating plant hormone balance is a key approach. Many halophilic/salt-tolerant microorganisms can synthesize auxins, directly stimulating plant cell division and root system development, thereby expanding the nutrient absorption area (Sagar et al., 2020; Patani et al., 2023). More importantly, they can produce 1-aminocyclopropane-1-carboxylate deaminase (Sagar et al., 2020; Yasmin et al., 2020). ACC is the precursor of ethylene biosynthesis. Salt stress induces massive ethylene accumulation in plants, accelerating senescence and inhibiting growth. ACC deaminase decomposes ACC into *α*-ketobutyric acid and ammonia, thereby reducing ethylene levels in plants and alleviating salt stress-induced growth inhibition (Shilev, 2020; Pandey and Gupta, 2020). Research demonstrates that ACC deaminase-producing bacterial strains exhibit particularly high efficacy in mitigating salt stress (Fatima et al., 2020; Nagaraju et al., 2021).

Secondly, enhanced plant nutrient acquisition serves as a direct growth-promoting mechanism. Halophilic/salt-tolerant microorganisms enhance soil availability of essential nutrients, including nitrogen, phosphorus, and iron, through nitrogen fixation, insoluble phosphate solubilization, and siderophore-mediated iron chelation, facilitating plant uptake and utilization (Sagar et al., 2020; Khumairah et al., 2022; Kusale et al., 2021). In saline soil, phosphorus availability is often reduced due to chemical fixation, where the application of salt-tolerant phosphate-solubilizing bacteria becomes particularly important (Dey et al., 2021). For instance, salt-tolerant Bacillus and Pseudomonas have demonstrated maintained phosphate-solubilizing activity under high-salinity conditions, thereby improving plant phosphorus nutrition (Amini Hajiabadi et al., 2021; Yan et al., 2024).

Thirdly, maintaining ion homeostasis and reducing sodium ion toxicity constitute the core mechanisms of salt tolerance. Under salt stress, plants encounter the dual challenge of excessive sodium ion accumulation and inhibited potassium ion uptake. Halophilic/salt-tolerant microorganisms can assist plants in maintaining a favorable K+/Na + ratio through multiple mechanisms. They may form a physical barrier on the root surface by secreting substances such as exopolysaccharides, thereby reducing the passive influx of sodium ions into roots (Thakur and Yadav, 2024; Fatima et al., 2020). Simultaneously, microorganisms induce plants to upregulate genes encoding ion transporters (e.g., SOS1 and NHX), enhancing the plants’ intrinsic capacity to compartmentalize sodium ions into vacuoles or efflux them from cells (Sampangi-Ramaiah et al., 2020; Saleem Asif et al., 2023). Studies confirm that plants inoculated with halophilic/salt-tolerant bacteria exhibit significantly reduced sodium ion content in shoots alongside increased potassium, calcium, and other beneficial ions, resulting in improved ionic balance (Masmoudi et al., 2021; Yasmin et al., 2020).

Fourth, enhancement of the antioxidant defense system to scavenge reactive oxygen species. Salt stress induces plants to produce excessive reactive oxygen species, resulting in membrane lipid peroxidation and cellular damage. Halophilic/salt-tolerant microorganisms induce plants to enhance the activity of antioxidant enzyme systems, including superoxide dismutase, peroxidase, catalase, and ascorbate peroxidase, thereby effectively scavenging ROS and protecting cellular structures (Ali et al., 2022; Tahira Yasmeen et al., 2020; Azeem et al., 2022). This induced systemic tolerance manifests not only in roots but also in shoots, indicating that microorganisms activate the plant’s comprehensive defense response (Yasmin et al., 2020).

Fifth, synthesis and accumulation of osmolytes. To cope with osmotic stress, microorganisms themselves and the plants they induce synthesize and accumulate compatible solutes such as proline, glycine betaine, soluble sugars, and polyols. These substances help maintain cellular turgor pressure and stabilize protein and membrane structures (Ayaz et al., 2022; Azeem et al., 2022). Some halophilic/salt-tolerant strains can directly provide these osmotic protectants to plants or stimulate their synthesis in plants (Fatima et al., 2020; Meena et al., 2020; Shahid et al., 2024). Moreover, exopolysaccharides produced by microorganisms serve as efficient osmotic buffers, forming a hydration layer in the rhizosphere to mitigate salt concentration fluctuations (Fatima et al., 2020; Chintan Kapadia et al., 2021).

Finally, biofilm formation and rhizosphere colonization constitute the physical foundation for their functional expression. Many effective salt-tolerant plant growth-promoting bacteria exhibit robust biofilm-forming capabilities (Tahira Yasmeen et al., 2020; Haroon et al., 2023). Biofilms enhance bacterial attachment to root surfaces, provide resistance against salt leaching, and create localized protected microenvironments conducive to material and signaling exchanges between microorganisms and plants (Thakur and Yadav, 2024). Successful rhizosphere colonization is the prerequisite for the implementation of all subsequent beneficial mechanisms (Muhammad Shoib Nawaz et al., 2020).

## 3 Applied research on the growth-promoting and salt-resistance effects of diverse halophilic/salt-tolerant microorganisms across various crop systems

The growth-promoting and salt-resistance effects of halophilic/salt-tolerant microorganisms have been extensively validated in multiple important food crops and cash crops, demonstrating diversity in application forms and strain sources.

Research has been most extensive in cereal crops. For wheat, salt-tolerant Bacillus and Planococcus strains isolated from halophyte rhizospheres significantly enhance germination rates, plant height, biomass, and grain yield under salt stress while improving grain and forage quality (Amini Hajiabadi et al., 2021). Microbial consortia composed of salt-tolerant strains enhanced wheat’s nitrogen and phosphorus uptake by boosting key soil enzyme activities, including urease and phosphatase, while effectively reducing leaf sodium ion content and oxidative damage (Muhammad Shabaan et al., 2022). For rice, endophytic fungi isolated from salt-tolerant varieties can impart salt tolerance to salt-sensitive varieties by regulating the expression of numerous abiotic stress-related genes (Sampangi-Ramaiah et al., 2020). Salt-tolerant rhizobia and plant growth-promoting rhizobacteria isolated from legume root nodules have also demonstrated efficacy in enhancing rice seed germination and seedling growth under salt stress (Mahmud et al., 2023). The application of composite biofertilizers optimizes the microbial community structure of salinized rice soils, improves soil available nutrients, and ultimately increases rice yield (Shan et al., 2023). In maize, salt-tolerant Bacillus and Pseudomonas strains exhibiting ACC deaminase activity significantly alleviated salt stress-induced growth inhibition and increased yield by enhancing photosynthetic efficiency, antioxidant enzyme activity, and osmolyte accumulation (Ali et al., 2022; Ali et al., 2023). The combined application of nanomaterials and salt-tolerant plant growth-promoting rhizobacteria synergistically enhances maize salt tolerance and yield by regulating ROS homeostasis and enriching beneficial rhizosphere microorganisms (Liu et al., 2022).

In vegetable and horticultural crops, the tomato serves as a typical model in research. Multiple halophilic/salt-tolerant Bacillus and Pseudomonas strains have been demonstrated to effectively promote growth, increase biomass, and improve physiological status in Tomato plants under salt stress through mechanisms including the production of plant growth-promoting substances, induction of antioxidant defense, and maintenance of ion homeostasis (Patani et al., 2023; Masmoudi et al., 2021; Pérez‐Rodriguez et al., 2022). Co-inoculation with endophytic fungi and arbuscular mycorrhizal fungi has also been shown to enhance drought and salt tolerance in Tomato and cucumber by regulating osmotic substances and antioxidant enzymes (Hosseyni et al., 2021). In soybean, salt-tolerant Pseudomonas and Bacillus mitigate salt stress damage by systematically regulating ion transport, antioxidant enzyme levels, and proline and malondialdehyde content (Khan et al., 2021; Yasmin et al., 2020). Microbial communities isolated from wild soybean root nodules offer clues for identifying salt-tolerant symbiotic bacteria (Yang Y. et al., 2020). Other crops, including pepper (Yañez-Yazlle et al., 2021), cucumber (Hosseyni et al., 2021; Fotoohiyan et al., 2024), lettuce, and sunflower (Szymańska et al., 2022), also demonstrate improved growth parameters and reduced salt stress damage following inoculation with salt-tolerant microorganisms.

Research on legumes and specialty crops, particularly chickpeas, has demonstrated that inoculation with ACC deaminase-producing salt-tolerant Bacillus strains not only enhances chickpea yield in saline soil but also alleviates oxidative stress by up-regulating antioxidant enzyme gene expression and promoting osmolyte accumulation (Nagaraju et al., 2021; Haroon et al., 2023; Basu et al., 2023). For common bean (*Phaseolus vulgaris*), inoculation with an exopolysaccharide-producing salt-tolerant rhizobium significantly improved growth and physiological adaptability under salt stress by enhancing ion balance, antioxidant enzyme activity, and osmolyte accumulation (Shahid et al., 2024). In quinoa, inoculation with endophytic Burkholderia stimulated growth and yield under salt stress by enhancing osmotic adjustment capacity and specific antioxidant enzyme activities (Yang A. et al., 2020). Salt-tolerant Bacillus and Pseudomonas isolated from mangroves alleviated salt stress in banana tissue-cultured seedlings by reducing reactive oxygen species accumulation and electrolyte leakage (Abdussabur et al., 2022).

Additionally, microbe-derived biostimulants demonstrate potential. For instance, plant hormone-rich extracts from Methylotrophic Actinomycetes culture filtrates effectively mitigated salt stress in wheat seedlings by enhancing antioxidant enzyme activities (Meena et al., 2020). The quorum-sensing signaling molecules, acyl-homoserine lactones, produced by salt-tolerant bacteria have also been found to promote root development in wheat under salt stress (Muhammad Shoib Nawaz et al., 2020).

## 4 Academic controversies and diverse perspectives

Although halophilic/salt-tolerant microorganisms demonstrate considerable potential in mitigating salt stress, academic controversies and unresolved perspectives persist in this field, primarily concerning the specificity of strain efficacy, complexities in combined applications, and the prioritization of underlying mechanisms.

Concerning strain efficacy specificity, a central debate revolves around the existence of a ‘universal’ high-performance strain. Some studies support strain-specific synergistic enhancement effects on particular crops or specific stress conditions. For example, one Pseudomonas strain significantly enhanced barley salt tolerance but showed minimal effects on lettuce and sunflower; whereas a Halomonas strain demonstrated universal growth promotion across all three crops (Szymańska et al., 2022). Similarly, in rice endophyte research, specific Fusarium fungi can confer salt tolerance to rice (Sampangi-Ramaiah et al., 2020), suggesting sophisticated interactive recognition between microorganisms and host plants. However, studies have also identified strains with broad-spectrum growth-promoting potential, such as certain Bacillus and Pseudomonas species isolated from halophytes, which exert beneficial effects on multiple crops including tomato and rice (Pérez‐Rodriguez et al., 2022; Sahu et al., 2023). This discrepancy between specificity and broad-spectrum effects may stem from interactions among multiple factors, including intrinsic microbial traits, host plant genotype, soil environmental context, and stress intensity.

Views also diverge regarding the synergistic effects and complexity of co-inoculation. Most studies suggest that combining microorganisms with different functions (such as PGPR and AMF) or microorganisms with other biostimulants (such as exopolysaccharides) can produce synergistic effects, alleviating salt stress more effectively than single inoculation (Chintan Kapadia et al., 2021; Diagne et al., 2020; de Carvalho Neta et al., 2024; Sánchez et al., 2023). For instance, co-inoculation of arbuscular mycorrhizal fungi with ACC deaminase-producing bacteria, through complementary advantages, more comprehensively improved the nutritional and physiological status of maize under salt stress (de Carvalho Neta et al., 2024). However, co-inoculation does not always produce positive synergistic effects. Competitive or antagonistic interactions may exist among microorganisms, and complex microbial interaction networks can lead to unpredictable outcomes (Muhammad Shoib Nawaz et al., 2020). Therefore, scientifically designing and optimizing microbial consortia to achieve stable and efficient synergistic effects remains a major challenge in practice.

There are also differing views regarding the relative effectiveness of endophytes versus rhizospheric bacteria. The conventional view holds that endophytes, due to their colonization within plant tissues, may engage in more direct and stable communication with the environment, thereby holding an advantage in inducing systemic resistance (Ben Slama et al., 2023). Some studies have indeed shown that certain endophytes significantly promote plant salt tolerance (Jhuma et al., 2021; Saleem Asif et al., 2023). However, other research indicates that many highly effective salt-tolerant plant growth-promoting bacteria isolated from rhizosphere soil can also function effectively through strong rhizosphere colonization and biofilm formation capabilities (Amini Hajiabadi et al., 2021; Tahira Yasmeen et al., 2020). In fact, the boundary between endophytes and rhizospheric bacteria is not always absolute, as many rhizospheric bacteria possess certain endophytic capabilities. The relative effectiveness of either group depends more on specific strain-plant combinations and environmental conditions than on simple origin-based classifications.

Moreover, while multiple potential mechanisms are known, the dominant pathways and activation sequences of signaling cascades remain unclear for specific strain-plant systems. For instance, whether ACC deaminase activity initially reduces ethylene levels, the antioxidant defense system activates first, or ion transport genes are induced primarily—the causal and temporal relationships among these events require further molecular-level dynamic investigations to elucidate (Roy et al., 2021).

## 5 Current research gaps and limitations

While significant progress has been achieved, current research also reveals critical gaps and limitations that constrain the large-scale application of halophilic/salt-tolerant microorganism technology from laboratory settings to field conditions.

First, mechanistic studies lack depth and systematic rigor. Most research remains confined to phenotypic observations and the measurement of a limited number of physiological and biochemical indicators (Shahid et al., 2023). Little is known about how microorganisms perceive salt signals, how the expression of beneficial genes (such as ACC deaminase genes and osmolyte synthesis gene clusters) is regulated by salinity, or the complete signaling dialogue network between microorganisms and plants (e.g., hormonal cross-talk, small RNA-mediated regulation) (Roy et al., 2021; Wu et al., 2024). Although genomic analyses have revealed that certain strains harbor abundant plant growth-promoting and stress-resistance genes (Wu et al., 2024; Zamanzadeh-Nasrabadi et al., 2023), research on functional validation and regulation of these genes under symbiotic conditions remains inadequate. A concrete illustration of this imbalance is that, whereas the myo-inositol metabolic pathway through which *Bacillus* sp. strain RA confers salt tolerance to tomato has been resolved at the gene and metabolite level (Wu et al., 2024), comparable pathway-level dissection is still lacking for the great majority of reported strain-crop systems, which are supported mainly by correlative physiological and biochemical data rather than by causal genetic evidence.

Second, the field application effects exhibit inconsistent and unpredictable outcomes. Numerous studies conducted under controlled greenhouse or potted conditions (Muhammad Shabaan et al., 2022) often yield results that are difficult to replicate in complex and variable field environments. Factors such as soil type, climatic conditions, farming practices, and competition from indigenous microbial communities can significantly impact the colonization success rate and functional expression of inoculated strains (Sagar et al., 2022). Ensuring the stable survival and expected functionality of introduced microorganisms within complex field ecological niches constitutes the primary bottleneck in practical applications. A further, often-overlooked tension deserves explicit attention. Although rigorous long-term, large-scale field validations of halophilic/salt-tolerant inoculants remain remarkably scarce in the peer-reviewed literature, PGPR-based biofertilizers are already produced and applied at commercial scale in several agricultural systems, including those of Brazil, India, and parts of South and Southeast Asia. Several non-mutually-exclusive explanations may reconcile this apparent contradiction. First, much field-performance data are generated in-house by manufacturers and remain proprietary or unpublished, so the gap may reflect a publication gap rather than a true absence of evidence. Second, methodological constraints—pronounced inter-site and inter-season variability, the technical difficulty of tracking introduced strains against established indigenous communities, and the high cost of multi-year replicated trials—discourage the standardized field studies that academic peer review expects. Third, a probable publication bias favouring positive greenhouse or single-season results over neutral or negative field outcomes further distorts the published record. Recognizing this discrepancy matters in both directions: the commercial uptake of PGPR should not be read uncritically as proof of efficacy under saline field conditions, nor should the scarcity of published long-term trials be taken as evidence that such products are ineffective.

Third, assessments of long-term impacts on soil microecosystems are lacking. While most studies focus on the immediate effects of inoculation on target plants, few have assessed its long-term impacts on soil microbial community structure, diversity, and functionality (Shan et al., 2023). Could the introduction of exogenous microorganisms disrupt or suppress native beneficial microbial communities? Might their metabolic activities alter soil carbon-nitrogen cycling? These ecological safety concerns remain inadequately addressed and lack systematic investigation (Kumar et al., 2023).

Fourth, the safety assessment of strains poses potential risks. Some studies prioritize screening strains for their growth-promoting and salt-tolerant traits while inadequately assessing potential risks of plant or animal pathogenicity (Muhammad Shoib Nawaz et al., 2020). For instance, certain Aeromonas strains exhibit favorable growth-promoting characteristics, but their biosafety must undergo rigorous verification before consideration for application (Muhammad Shoib Nawaz et al., 2020). Applying safety-assessed microorganisms inadequately in agriculture may entail unknown ecological and health risks.

Fifth, the systematic and targeted exploration of microbial resources needs enhancement. Although numerous halophilic/salt-tolerant microorganisms have been isolated from various extreme environments (such as salt-affected lands, coastal zones, and halophytes), the screening strategies are sometimes relatively random and lack purpose-oriented screening targeting specific crops or particular stress mechanisms (e.g., efficient sodium exclusion, strong antioxidant induction) (John et al., 2023; Aizaz et al., 2023). Relatively little attention has been paid to important microbial groups beyond bacteria, such as fungi and actinomycetes (Hosseyni et al., 2021; Aizaz et al., 2023).

Finally, efficient microbial agent development and delivery technologies lag behind. Developing excellent laboratory strains into commercial microbial agents with long shelf-life, stable activity, ease of application, and cost-effectiveness is a key technical challenge for industrialization (AbuQamar et al., 2024). Existing inoculation methods (such as seed coating, soil drenching, and root dipping) exhibit considerable variability in efficacy across different conditions, necessitating optimization and innovation (Pandey and Gupta, 2020). Closely related is the limited availability of independent, long-term performance data for commercial inoculants. Publicly accessible records of how marketed products behave across multiple growing seasons, soil types, and salinity gradients are scarce—partly because such data are frequently retained as proprietary commercial information with restricted access, partly because field variability makes consistent, reportable performance difficult to demonstrate, and partly because of the publication bias noted above. Consequently, researchers and end-users alike often lack the transparent, comparable efficacy data needed to distinguish genuinely robust products from those whose benefits are context-dependent or transient, which in turn slows independent verification and erodes confidence in the technology.

## 6 Conclusion and future potential directions

In summary, halophilic/salt-tolerant microorganisms demonstrate significant application potential in alleviating plant salt stress and promoting growth through multiple synergistic mechanisms, including modulating plant hormones, improving nutrient uptake, maintaining ion homeostasis, enhancing antioxidant defense, and synthesizing osmolytes. Research has covered various crops including rice, wheat, maize, tomato, and legumes, and has begun exploring more complex application modes such as microbial consortia and microbe-substance synergies. However, this field still faces challenges including incomplete understanding of mechanisms, inconsistent field performance, and unclear ecological risks.

Future research should focus on the following potential directions to advance scientific progress and technological translation in this field:

First, deepen research on molecular interaction mechanisms and systems biology. Utilizing multi-omics technologies combined with genetic and molecular biological approaches, future research should thoroughly analyze the entire chain of molecular dialogue mechanisms—from recognition and colonization to systemic resistance induction—between key microbial strains and host plants under salt stress (Roy et al., 2021; Wu et al., 2024). This section focuses on elucidating how microbial effector molecules regulate transcriptomic, proteomic, and metabolomic reprogramming in plants, utilizing plant mutants or microbial gene-knockout strains to validate core gene functions (Saleem Asif et al., 2023; Vaishnav et al., 2020). This will establish the theoretical foundation for designing rational and targeted microbial inoculation strategies.

Second, develop precision microbiome engineering-based regulation strategies. Future applications should extend beyond single or limited-strain inoculations toward rationally designed approaches based on microbiome studies (Ben Slama et al., 2023). By investigating the structure and function of core microbiomes in salt-tolerant plants, key ‘keystone’ species or functional gene clusters can be identified. Through soil management, companion planting, or ‘prebiotic’ supplementation, beneficial microbial communities in soil and the rhizosphere may be directionally cultivated and modulated to construct stress-resilient, productive plant-microbe symbiotic systems (Yan et al., 2024; Liu et al., 2022; Wang et al., 2023).

Third, optimize microbial inoculation techniques and intelligent delivery systems. Develop novel microbial agents, such as encapsulation technologies based on biofilms, microcapsules, or nanocarriers, to enhance the survival rate and stability of inoculants during storage and application (Thakur and Yadav, 2024). Explore microbial delivery methods integrated with modern agricultural technologies (e.g., drip fertigation, precision agriculture). Concurrently, develop molecular tracking technologies capable of real-time monitoring of inoculant strain colonization and functional gene expression, thereby providing data for efficacy evaluation and field management.

Fourth, strengthen field validation studies and ecological safety assessments. Long-term, multi-site field trials must be conducted to systematically evaluate the efficacy stability and economic feasibility of different microbial products across diverse soil-climate-crop systems (Kusale et al., 2021). Establish standardized ecological risk assessment systems to comprehensively evaluate the long-term impacts of inoculated microorganisms on non-target organisms, soil microbial network structure, nutrient cycling processes, and potential gene horizontal transfer (Kumar et al., 2023; Muhammad Shoib Nawaz et al., 2020). This is a prerequisite for ensuring the environmental safety of microbial agricultural technologies, gaining public acceptance, and obtaining regulatory approval.

Fifth, explore novel microbial resources and functional metabolites. Continue to mine microbial resources with unique growth-promoting and stress-resistance mechanisms from extreme environments, rare salt-tolerant plants, and underutilized microbial taxa (e.g., Archaea and Fungi) (John et al., 2023; Aizaz et al., 2023). Concurrently, enhance research on the isolation, identification, and functionality of bioactive metabolites derived from microorganisms—such as novel quorum-sensing molecules, volatile organic compounds, polysaccharides, and lipopeptides. These metabolites can be developed directly as Biostimulants or biopesticides (Meena et al., 2020; Sánchez et al., 2023; Shahid et al., 2023).

Through sustained efforts in these areas, Halophilic/salt-tolerant microorganism technology holds promise as a robust solution for addressing soil salinization challenges while promoting global food security and sustainable agricultural development.
